# Efficacy of the Virtual Reality Intervention *VR FestLab* on Alcohol Refusal Self-Efficacy: A Cluster-Randomized Controlled Trial

**DOI:** 10.3390/ijerph19063293

**Published:** 2022-03-10

**Authors:** Julie Dalgaard Guldager, Satayesh Lavasani Kjær, Ulrike Grittner, Christiane Stock

**Affiliations:** 1 Unit for Health Promotion Research, Department of Public Health, University of Southern Denmark, Degnevej 14, DK6705 Esbjerg, Denmark; jguldager@health.sdu.dk (J.D.G.); satakjaer@health.sdu.dk (S.L.K.); 2 Department of Physiotherapy, University College South Denmark, Degnevej 16, DK6705 Esbjerg, Denmark; 3 Institute of Biometry and Clinical Epidemiology, Charité–Universitätsmedizin Berlin, Corporate Member of Freie Universität Berlin, Humboldt-Universität zu Berlin, and Berlin Institute of Health, Charitéplatz 1, 10117 Berlin, Germany; ulrike.grittner@charite.de; 4 Institute for Health and Nursing Science, Charité–Universitätsmedizin Berlin, Corporate Member of Freie Universität Berlin, Humboldt-Universität zu Berlin, and Berlin Institute of Health, Augustenburger Platz 1, 13353 Berlin, Germany

**Keywords:** adolescents, virtual reality, alcohol, alcohol prevention, drinking refusal self-efficacy, peer pressure, cluster-RCT, intervention, school-based prevention

## Abstract

It is currently unknown whether a virtual social environment can support young people in building their skills to overcome peer pressure when offered alcohol. This study evaluated the efficacy of the newly developed virtual reality simulation game *VR FestLab* on the refusal self-efficacy regarding social pressures to drink of Danish male and female students aged 15–18. *VR FestLab* features a party setting where adolescents can “steer” their own party experience. Eleven schools were included in a cluster-randomized controlled trial and allocated to either the intervention (*n* = 181) or the active control group (*n* = 191). Students in intervention schools played *VR FestLab*, while those in the control group played the VR game *Oculus Quest—First Steps*. The primary outcome measure was the social pressure subscale of the drinking refusal self-efficacy scale (DRSEQ-RA). The intervention effects were measured immediately after the intervention/control session (T1) and after a 6-week follow-up (T2). Data were examined using linear mixed regression models. Our study did not demonstrate a significant effect of drinking refusal self-efficacy at T1. For all secondary outcomes, we observed no substantial differences between the intervention and control groups. This study provides new insights into the feasibility and effectiveness of an innovative virtual reality alcohol prevention tool. *VR FestLab* can be an innovative and promising contribution to complement existing school-based alcohol prevention, but more research is needed to improve its effectiveness.

## 1. Introduction

Denmark is among the countries with the highest rate of risky alcohol use in Europe [1]. A recent Danish study shows that 80% of 15–25-year-olds have experienced being drunk at least once [2]. Alcohol use is a major risk factor for a number of diseases and contributes as a significant factor to homicides, suicides, and motor vehicle fatalities [3]. Moreover, the social development of adolescents and young adults is often negatively affected by alcohol and other substance use. For example, unwanted pregnancy, injuries, school failure, and other problems are associated with substance use [4,5]. Adolescents who begin drinking at a younger age also tend to have lower self-esteem, be less resistant to peer pressure, and display anti-social behaviors [6].

It is well-accepted that prevention programs are important because the earlier adolescents start using alcohol, the more likely it is that they will abuse alcohol later in life [7,8,9]. Understanding what leads children and adolescents to begin using alcohol is important for developing effective programs. Peer pressure has been identified as one of the most common reasons for adolescents taking up and continuing the consumption of alcohol [10,11,12]. Although the conclusions from reviews of the effectiveness of social influence prevention programs are not entirely consistent, the majority of the evidence is favorable [3,13]. Moreover, the role of refusal self-efficacy in the development and maintenance of drinking behavior, including in situations of peer pressure, is well-established [14,15].

Thus, many prevention programs conducted in schools comprise elements of refusal skills training, with a particular focus on resisting peer pressure [16]. An adolescent’s decision to drink alcohol or use drugs is believed to depend on his or her ability to resist the social pressures common in early adolescence [17]. Refusal skills training aims at inoculation against the social influences of adolescence based on inoculation theory [18] by teaching adolescents how to recognize social pressure from peers (e.g., a potential new friend offers a drink), older siblings, or the media. Refusal skills training also entails how to cope with high-pressure situations by developing social skills to refuse explicit alcohol offers without experiencing negative social consequences (e.g., losing friends, being stereotyped by peers) [17]. The development of adequate refusal skills is the main goal of refusal skills trainings that are often featured in prevention programs. A review showed that refusal skills programs show favorable effects on alcohol drinking in older adolescents but may not be effective for younger age groups in early adolescence [19]. A sensitive period for the development of refusal skills seems to be the middle adolescence (14–18 years) which, according to Steinberg and Monahan [20], is “an especially significant period for the development of the capacity to stand up for what one believes and resist the pressures of one’s peers to do otherwise” (p. 1531).

Traditional training of refusal skills mostly uses role-play, guided practice, or role modeling to provide learners with practical experience. Virtual reality (VR) is an emerging educational tool [21,22] that allows us to simulate a virtual learning environment that closely approximates reality [23]. The convincing nature of characters and environments in VR simulations can improve learning and encourage adolescents to strengthen their own levels of knowledge and skills. This offers unique opportunities to provide learners with similar-to-real-life experiences of reacting positively and in socially accepted ways to avoid social pressure to drink. VR simulations are engaging for adolescents and have been demonstrated to achieve initial teaching successes [22,24]. Virtual reality allows for a novel approach to teaching refusal-efficacy skills as well as favorable alcohol expectancies and enables health educators to immerse adolescents in environments of alcohol consumption and risk-taking without the physical dangers that these behaviors may lead to in real life [25]. 

We assume that adolescents will benefit from using such tools because it is engaging, fun to use, and effective at developing relevant life skills and favorable alcohol expectancies. However, there exist only very few similar virtual reality applications for use in health promotion and preventive interventions worldwide. A recently developed VR e-cigarette prevention game for adolescents entitled *Invite only VR*, was found to be both useful and insightful. The study found that adolescents had significantly improved their knowledge regarding e-cigarettes and nicotine addiction, perception of harm, and social perception about e-cigarette use 6 months from baseline compared with the control group. Furthermore, the participants expressed high ratings of gameplay experience and satisfaction [26,27].

Another earlier developed VR avatar-based game simulation, *DRAMA-RAMA^TM^*, was shown to be effective in strengthening the resistance skills of Hispanic early adolescent girls to avoid being pressured into risky behavior, such as early sexual behavior, compared with an active control game experience [28].

Within the area of VR simulations targeting alcohol prevention for adolescents, a recent review [29] identified only two studies besides the present authors’ *VR FestLab* that met the inclusion criteria. An American project exposed adolescents to a VR party including simulated substance use and sexual risk-taking cues and found an increase in physiological arousal among users [30]. The Australian *Blurred Minds Program* is the first application of VR-based alcohol refusal self-efficacy training. *VR House Party* is one of the game- and activity-based components of the five-lesson program and quantitative and qualitative testing showed that this was well-received by adolescents [25]. An effectiveness trial of the entire *Blurred Minds Program* demonstrated preventive effects on adolescents’ knowledge, attitudes, and intention to drink, but separate tests on the effects of *VR House Party* on alcohol refusal skills were not conducted in this trial [25]. Therefore, to the best of our knowledge no VR-based alcohol resistance tool has been tested before in a controlled trial with respect to its efficacy at enhancing drinking refusal self-efficacy [21].

### Aims and Objectives

This study evaluated the efficacy of the *VR FestLab* game aimed at improving the refusal self-efficacy of adolescents (15–18 years) who face social pressures to drink alcohol via a cluster-randomized controlled trial. The primary outcome was drinking refusal self-efficacy (DRSEQ-RA). The secondary objective was to test the effects of the game on encouraging adolescents to exhibit more responsible behavior in party situations. The main hypothesis is that adolescents who experience *VR FestLab* will attain a higher drinking refusal self-efficacy score immediately after the intervention at the follow-up assessment (T1) compared with those in the active control group. The secondary hypothesis is that adolescents in the intervention group compared with those in the control group are expected to have higher scores in drinking refusal self-efficacy six weeks later at the second follow-up (T2). Further, adolescents in the intervention group compared with those in the control group are expected to have higher scores in drinking refusal self-efficacy, drug refusal skills, knowledge/awareness, communication skills, social support willingness and lower levels of susceptibility to peer pressure, and positive outcome expectations at T1 and T2.

## 2. Materials and Methods

### 2.1. Study Design

The efficacy of *VR FestLab* was tested in a cluster-randomized controlled trial (cluster-RCT) (registration number ISRCTN11768445). Schools were randomly assigned (1:1) to the intervention/active control group before the collection of baseline data. 

### 2.2. Participants

For the trial, 13 Danish public/boarding schools were recruited in the Region of Southern Denmark. Boarding schools ensure a better geographical coverage of study participants, since their students typically have home addresses and social backgrounds in different parts of Denmark. Schools were invited to participate via an email to the school principal. To increase the willingness of schools to participate, a session with *VR FestLab* was offered to control schools after the trial period.

Participants were recruited through principals and/or teachers at the schools. For inclusion in the study, all students aged 15–18 were eligible. The exclusion criterium was insufficient knowledge of Danish to understand typical everyday conversations. The students and their parents received an information brochure about the study and were asked to indicate their willingness to participate within two weeks. Written consent was obtained from all students (in Denmark, informed consent from parents is only required for children younger than 15 years). All data were collected anonymously and treated confidentially. 

### 2.3. Data Collection

Data collection took place at three time points. The baseline assessment (T0) conducted before the intervention, but after school randomization, comprised a questionnaire for the students (see details in the next section). Immediately after the intervention/active control session the follow-up assessment (T1) was completed and six weeks later, the second follow-up (T2) data collection was completed with the same questionnaire as T1 (except for the gameplay experience questions). To link each participant’s responses from the three different time points, an individual anonymous code was generated based on class code, number of older brothers, date of birth, first letter in mother’s name, and two first letters of eye color, following procedures outlined in Rundle-Thiele et al. [31]. Unmatched codes were excluded from the analysis.

### 2.4. VR FestLab: An Alcohol Prevention Intervention

*VR FestLab* was developed for school-based alcohol prevention and was, after data collection, made available as a free Danish smartphone app. It was developed using a co-creation approach with young people and other stakeholders. Details of the intervention development are described elsewhere [32,33]. 

We followed the taxonomy of the Behavior Change Wheel [34] (see the Appendix A for a figure) to describe the behavior change functions incorporated into *VR FestLab* and to analyze the expected changes regarding the intermediate factors of capability, opportunity, and motivation that affect the behavioral outcome resisting peer pressure to drink. *VR FestLab* applies the following practical methods for the different behavior change functions:

Education: While playing *VR FestLab* the user is confronted with several behavioral options, where peers encourage the user to choose either to drink alcohol or soft drinks/water, dance, play, or interact with others. The user´s decision to drink alcohol, the type of drinks (low or high in alcohol concentration), and the time between consuming alcoholic drinks is computed by underlying software to provide visual feedback on blood alcohol concentration (BAC). A concrete level of BAC is not displayed, but a bar displays the relative BAC level from zero to maximum (end of game and fading out) level. In addition, bubbles are displayed with increasing BAC. This aims to enhance the user’s knowledge and awareness regarding the effects of different alcoholic drinks on the physical state of the body.

Training: The behavioral options are related to communication options, i.e., how to respond to peer pressure. While “yes” type answers to offers of alcoholic drinks lead to progressively more devastating effects for the user (short duration of the game, getting sick and throwing up), the user can train more complex communication and behavioral options by re-setting the game. If options chosen are “wrong”, players can replay and engage with a more positive party experience and positive feedback from peers the next day (screen shots of text messages displaying what peers think about their behavior at the party).

Modelling: Throughout the game, the user experiences peers with positive and responsible behavior regarding alcohol drinking. These role-models either do not drink at all but are still socially attractive and/or support their friends if they are intoxicated. Through learning from role-models, social opportunities are created to act towards the modelled communication and behavior of responsible alcohol-related practice.

Coercion/incentivization: *VR FestLab* demonstrates the social costs of over-consumption of alcohol through a variety of consequences that the user potentially experiences, such as not being able to flirt with attractive peers due to high alcohol level, not being able to go to the party at all, or being confronted with negative peer feedback via texting the next day. On the contrary, abstinence or moderate drinking is incentivized by more behavioral and flirt options, a longer party experience, and positive peer texting. Throughout such coercion/incentivization methods, *VR FestLab* is expected to decrease the susceptibility to peer pressure faced by adolescents and to decrease positive outcome expectations towards more realistic consequences of drinking alcohol in party situations. A figure summarizing the theory of change and behavior functions of *VR FestLab* can be found in the Appendix A.

#### 2.4.1. Gameplay Session

The gameplay session with *VR FestLab* or the control game *Oculus Quest—First Steps* took place during an in-class teaching session. The session started with a gameplay introduction and exploration phase of about 45 min. Thereafter, a structured reflection of the experiences was moderated by a trained study assistant. The reflection phase aimed at providing the opportunity for a structured sharing of experiences in class rather than an informal chat during breaks. 

For the introduction, a trained study assistant started either the intervention or the control game and instructed the entire class. *Oculus Quest* VR devices were used for the trial with the intervention and control games installed. When the teaching session started, students were instructed how to wear the VR devices and how to navigate during the game play with head movements. The gameplay was limited to 15 min for each participant, but participants could finish at any time if they felt uncomfortable. The class was divided into groups of a maximum of 13 students who played the game at any one time. The subsequent reflection phase differed between the intervention and control schools.

#### 2.4.2. Intervention Schools

For the reflection phase, student tasks were prepared, which offered students in groups of 4–6 a choice between one of three scenarios related to *VR FestLab* (e.g., to be at a party while not wanting to drink alcohol, such as one of the *VR FestLab* characters) and in their groups they discussed questions such as “how do you behave?” and “what do you think other peers should do?” for up to 15 min. Thereafter, the study assistant facilitated a discussion of the students´ experiences and reflections in class for up to 15 min.

#### 2.4.3. Active Control Intervention

The game *Oculus Quest—First Steps* used in the active control classes demonstrates the interactive options that VR offers in an enjoyable and entertaining way (such as dancing with an avatar or selecting tools for a shooting game). The game does not offer educational content except for learning how games using VR work in principle. The reflection phase was structured in the same way as for the intervention classes, but reflections were about the possibilities of using new technologies at school with respect to questions such as “what are your ideas about using VR at school?” and “who could benefit from teaching based on VR or other new technologies?”. After completing the second follow-up data collection, a session with *VR FestLab* and the ensuing classroom discussion was offered to the control schools.

### 2.5. Questionnaires and Outcomes

The content of the questionnaire, time of data collection, and original and collapsed response categories are reported in Table 1. Details of the origin of measures were reported in a statistical analysis plan published at figshare.com [35]. 

**Table 1:** Questionnaire variables and response categories.

Variable	Data Collection Time	Question	Original Response Categories	Collapsed Response Categories
Sex	T0	Are you a girl or a boy? (State what you most identify as right now)	Boy/Girl	None
Age	T0	How old are you?		
Perceived family affluence	T0	How well-off do you think your family is?	Very well-off/Quite well-off/Average/ Not so well-off/ Not at all well-off	Low to medium (Not at all well-off/Not so well-off/ average) High (Quite well-off/ Very well-off)
Lifetime binge drinking	T0	Have you ever drank five or more drinks on a single occasion?	Yes/No	None
Sensation seeking	T0	Eight-item Brief Sensation-Seeking Scale (BSSS)	Five-point Likert scale from disagree strongly to agree strongly	Sum score
Potential adverse effects	T1	Did you experience any side effects when trying *VR FestLab*?	Open question	Responses grouped into the categories of; no side effects, cybersickness (from symptoms of cybersickness [36,37]), physical distress from wearing the VR equipment [38], and other symptoms/not specified
Drinking refusal self-efficacy	T0/T1/T2	Five-item Social Pressure subscale of the Drinking Refusal Self-Efficacy Questionnaire (DRSEQ-RA)	Six-point Likert scale from “I am very sure I could NOT resist drinking” to “I am very sure I could resist drinking”	Sum score
Drug refusal skills	T0/T1/T2	Seven-item drug refusal skills subscale from the Brief Assessment life skills Training Tool	Not refuse/likely not refuse/likely refuse/refuse	Sum score
Knowledge/Awareness of blood alcohol concentration	T0/T1/T2	“It is easy for me to estimate my own alcohol tolerance”, “I know how much alcohol I can drink before I get drunk”	Five-point Likert scale from disagree strongly to agree strongly	Sum score
Communication skills	T0/T1/T2	“If my best friends want me to drink beer with them and I don’t want to, I have ways to say no” “If someone offers me a drink of alcohol and I say “no”, I can make them take “no” for an answer”	*Same*	Sum score
Social support willingness	T0/T1/T2	“If someone is really drunk or sick at a party, the best thing to do is…”	Let him or her recover alone (0 point)/Help him or her to recover (1 point)/Ask an adult for help (1 point)/Call his or her parents (1 point)	Sum score for each item ticked
Susceptibility to peer pressure	T0/T1/T2	“If I am at a party and my friends are drinking alcohol, I would feel left out if I were not drinking alcohol.”	*Same*	Item score
Outcome expectations	T0/T1/T2	How much do you agree that the following happens to you if you drink alcohol? I become more fun/more happy I become more extroverted I become more confident I forget my problems	Five-point Likert scale from disagree strongly to agree strongly	Sum score

#### 2.5.1. Content of the Baseline Questionnaire (T0)

##### Primary Outcome Measure

Resistance towards peer pressure to drink was measured using the Social Pressure subscale of the adapted version of DRSEQ-RA with a Cronbach´s alpha of 0.87. The subscale for the DRSEQ-RA was shown to be correlated with alcohol consumption to establish the concurrent validity of the revised scoring method [39]. The Cronbach´s alpha was 0.88 in our sample.

##### Secondary Outcome Measures

Drug refusal skills were measured using the subscale on Drug Refusal Skills from the Brief Assessment Tool of the Life Skills Training [40] with a Cronbach´s alpha of 0.85 [40]. The Cronbach´s alpha was 0.62 in our sample.

**Figure A1:**
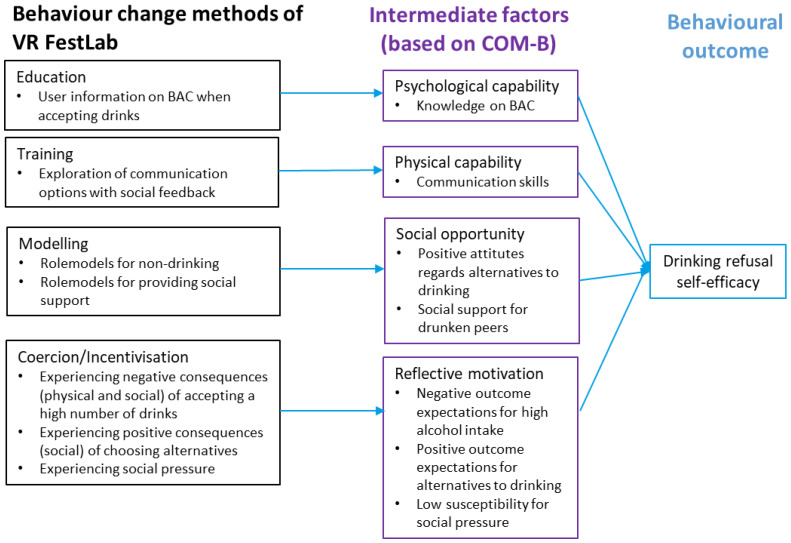
Theory of change of *VR FestLab* using the Behavior Change Wheel categorization based on Michi et al. [34].

We measured the intermediate effects of the behavior change functions education, training, modelling and coercion/incentivization (as described in Figure A1 in the Appendix A) on the following secondary outcomes as potential moderating variables: knowledge/Awareness of blood alcohol concentration (own addition), communication skills (from the Alcohol Misuse Prevention Knowledge Questionnaire [41]), susceptibility to peer pressure (from the SPP index [42]), social support willingness (own addition), and outcome expectations (from the Danish Youth profile [43]). 

Demographic information on age, sex, and family socioeconomic status was collected (from the Health Behavior in School-aged Children study [44]). Additionally, we asked students about their alcohol experience (whether they had ever drank five or more drinks on one occasion based on lifetime measures from ESPAD [45]). All participants were asked to respond to the *Sensation Seeking Scale* for adolescents with a Cronbach´s alpha of 0.76 [46] (it was also 0.76 in our sample).

#### 2.5.2. Content of the Follow-Up Questionnaires

The follow-up questionnaire contained all questions related to the primary and the secondary outcomes from the baseline questionnaire. In the follow-up questionnaire at T1, we also asked students an open question regarding potential adverse effects, such as cyber sickness [47]. 

#### 2.5.3. Questionnaire Development and Validation

The questionnaires were developed using the English versions of the respective scales as described above, where no Danish versions existed. The project team developed our own items for those secondary outcomes for which no scales existed in the literature. The English questionnaires were forward and backward translated to Danish following the WHO Process of translation and adaptation of instruments [48] and pre-tested in 31 students to determine the psychometric characteristics. 

### 2.6. Sample Size Calculation

We used an estimated mean of the Social Pressure subscale of the DRSEQ-RA of 19.0 with 6.5 standard deviation based on previous studies [14,49]. No intervention effect measures for DRSEQ-RA in similar intervention settings were available. However, we assumed that we would detect an intervention effect of at least 0.44 (Cohen’s d), which corresponds to a mean difference of 2.85 and a common standard deviation of 6.5 points. An intervention effect of similar size was determined in other studies as a relevant increase in refusal self-efficacy in adolescents [28]. Using STATA 15, a power calculation for a cluster-RCT and a two-sample t-test resulted in a sample size of 135 for the control group and 135 for the intervention group to detect an intervention effect of 0.44, assuming a power of 0.80 and using a two-sided alpha of 0.05. The sample size was calculated based on an estimated intra-class correlation for drinking refusal self-efficacy of 0.01 and 45 students per school. Taking an estimated attrition of 35% into account, we planned to recruit 420 participants for the trial (210 for each group). 

### 2.7. Randomisation and Blinding

The study coordinator enrolled the schools, which were randomly assigned (1:1) to intervention/active control group by an external statistician before baseline data was collected. The study coordinator was informed of each school’s status as intervention/active control group as close to the school visit as possible, and this status was disclosed to schools at the beginning of the session on the day of the school visit. Randomization was stratified by type of school (general public school/boarding school) and conducted by an independent statistician blinded to the identity of the schools. Due to the nature of the intervention, participants and the study assistants who led the session were not blinded for the intervention. The statistician assessing the outcomes was not blinded to intervention and control conditions but was external to the research group conducting the trial.

### 2.8. Statistical Analysis

IBM-SPSS for Windows v.28 and the statistical software R [50] were used to conduct the statistical analysis and a detailed analysis plan was published at figshare.com [35]. R packages tidyverse [51], mice [52], lme4 [53], emmeans [54], and metafor [55] were employed. Absolute and relative frequencies of students’ baseline characteristics in total and by group (intervention/control) are reported in Table 2. The means and standard deviations of primary and secondary outcomes by time point and group are reported in Table 3. Absolute and relative frequencies and 95% CI of potential adverse effects experienced by students in total and by group (intervention/control) are reported in Table 4. Missing values at follow up time points were estimated using multiple imputation by chained equations (as implemented in the R package mice), resulting in 30 imputed datasets. Estimation of missings was based on all baseline characteristics and all available outcome measures at each time point. Primary and secondary analyses were conducted within the full analysis set (intention to treat, ITT). Intervention efficacy (primary outcome analysis) was established with a linear mixed regression model (random intercept for schools) at follow-up T1 using a two-sided significance level of α = 0.05. This model tested group differences in the primary outcome (drinking refusal self-efficacy towards social pressures to drink) at T1 while adjusting for sex, age category, baseline value of drinking refusal self-efficacy, as well as interaction terms for intervention group by sex, intervention group by age group, and intervention group by baseline value (median split). Results are presented as marginal effects and 95% CI. Additional models were used to estimate subgroup differences between students who were lifetime binge drinkers vs. not lifetime binge drinkers, students with different baseline levels of sensation seeking, and high vs. low family affluence with the same covariates and additional interaction terms for these subgroups. For each secondary outcome, separate linear mixed models (random intercept models with random intercept for schools) were used with the particular outcome at T1 or T2 as dependent variables and group, age group, sex, and the particular baseline measure as covariates. The results are presented as marginal effects and 95% CI. Due to a mistake in the questionnaire, positive role models were not measured, which is a deviation from the statistical analysis plan [35].

All secondary analyses were conducted using the full analysis set with multiple imputed data in the case of missing values. All secondary analyses were performed in an exploratory framework. The interpretation of the results is based on the effect estimates and 95%CIs. 

## 3. Results

### 3.1. Participant Flow and Recruitment

Participating schools were recruited from May to October 2020. School visits were conducted between August and December 2020 and again from April to May 2021 (the gap was due to COVID-19 school closings). Follow-up data were collected online 5 to 6 weeks after school visits.

**Figure 1:**
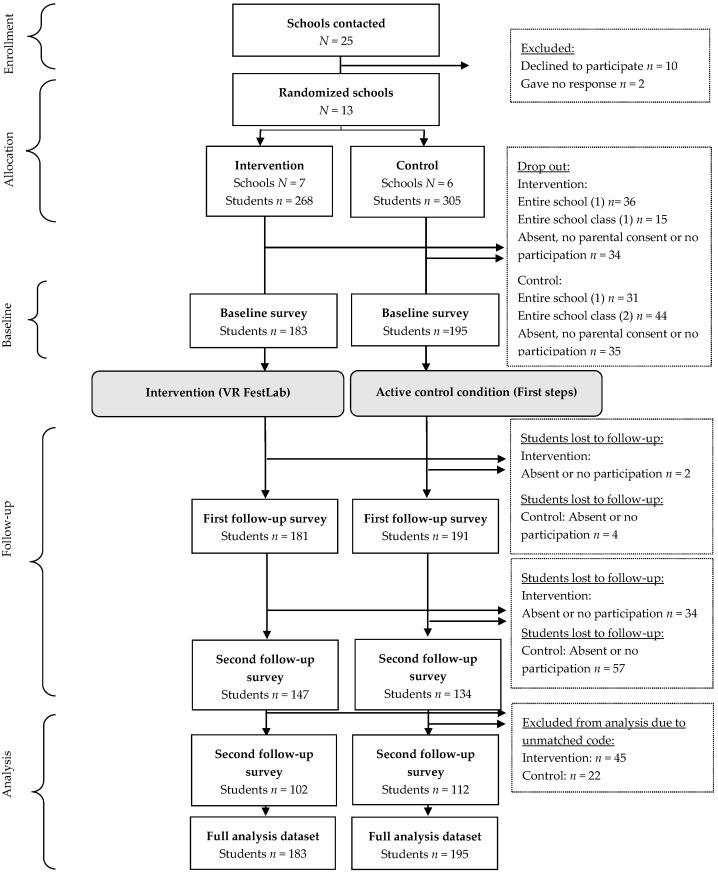
Participant flow through the trial.

The participant flow throughout the trial and number analyzed are depicted in Figure 1. In total, 378 students from 11 schools completed the baseline survey (T0) with 183 (48.4%) in the intervention group and 195 (51.6%) in the control group. A total of 372 students (98.4%) completed the first follow-up assessment (T1) (intervention *n* = 181, control *n* = 191) and 214 (56.6%) students completed the second follow-up (T2). 

### 3.2. Student Characteristics

Table 2 depicts information about the characteristics of the participating students. A total of 192/379 (50.8%) of the students were female, 317/378 (83.9%) were 15–16 years old with a higher proportion of students in the intervention group being 16 years old or younger compared with the intervention group. A total of 319/378 (84.4%) had low to medium perceived family affluence. Concerning lifetime alcohol, the majority of students (277/378, 73.3%) reported having partaken in binge drinking.

### 3.3. Outcome Measures at T0, T1, and T2

The mean values and SD for primary and secondary outcomes at T0, T1, and T2 stratified by intervention and control group can be found in Table 3. For the intervention and control group, drinking refusal self-efficacy increased from T0 to T1 and from T1 to T2. Drug refusal skills were for both groups unchanged from T0 to T1 but increased from T1 to T2. For both the intervention and control group, knowledge about blood alcohol concentration, communication skills, social support willingness, and susceptibility to peer pressure were unchanged from T0 to T1 and from T1 to T2.

### 3.4. Multilevel Intervention Effects for the Primary Outcome and Sub-Group Effects at First Follow-Up

**Figure 2:**
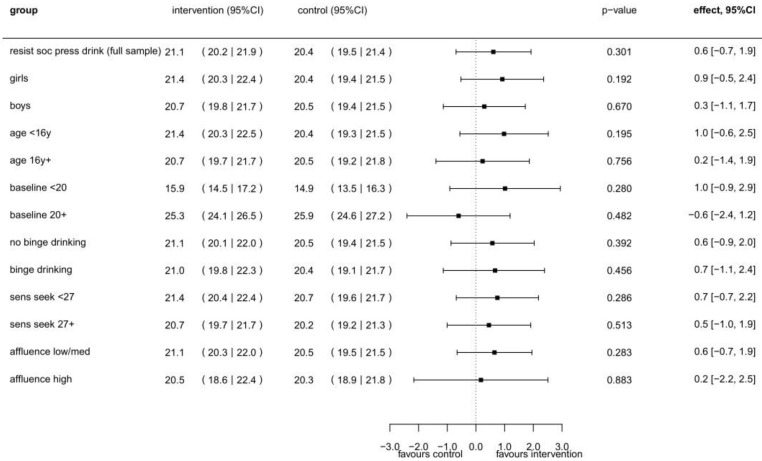
Intervention effects for drinking refusal self-efficacy (social pressure subscale of DRSEQ-RA) at T1 (*n* = 378), in total sample and by sex, age, family wealth, baseline value, lifetime binge drinking, and sensation seeking * based on linear mixed models ^#^. * Variable names in graph: drinking refusal self-efficacy: “resist soc press drink”; baseline value of DRSEQ-RA less than or over the mean of 20: “baseline <20/20+”, lifetime binge drinking: “lifetime binge”; sensation seeking with values less than or over the mean of 27: “sens seek <27/27+”; family affluence: “affluence”. ^#^ The model for the first five estimates is adjusted for sex, age, and baseline value. Additional separate models were used for subgroup analyses of lifetime binge drinking, sensation seeking, and family affluence. All these models were adjusted for age, sex, and baseline value.

For the primary outcome of drinking refusal self-efficacy, a small effect of 0.6 favoring the intervention was found, which was not statistically significant (95% CI: −0.7–1.9) (see Figure 2). The effect was somewhat higher for girls than for boys, for students under the age of 16 than for older students, for students with baseline data of drinking refusal self-efficacy below median than for those with higher baseline levels, and for students with low/medium family affluence than for those with high family affluence. 

### 3.5. Multilevel Intervention Effects for Secondary Outcomes at First Follow-Up

**Figure 3:**
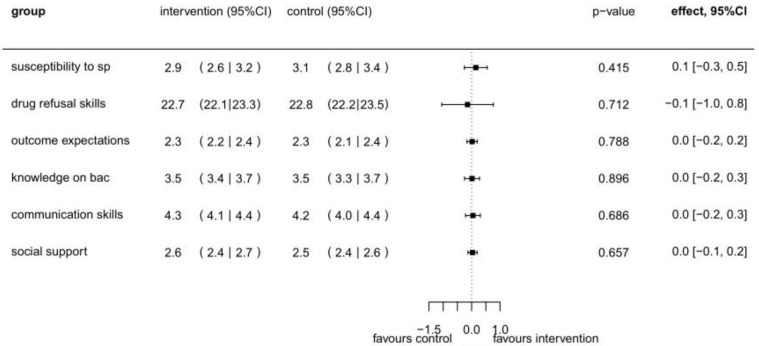
Intervention effects for secondary outcomes (susceptibility to social pressure, drug refusal skills, outcome expectation, knowledge on blood alcohol concentration, communication skills, and social support) * at T1 (*n* = 378) based on linear mixed models ^#^. * Labels used in graph: social pressure: “sp”; blood alcohol concentration: “bac”. ^#^ Separate regression models were used for each outcome. The models are adjusted for sex, age, and the particular baseline value.

For the secondary outcomes, susceptibility to social pressure, drug refusal skills, outcome expectation, knowledge of blood alcohol concentration, communication skills, and social support, no substantial differences between the intervention and control groups were observed at T1 (see Figure 3).

### 3.6. Multilevel Intervention Effects at Second Follow-Up

**Figure 4:**
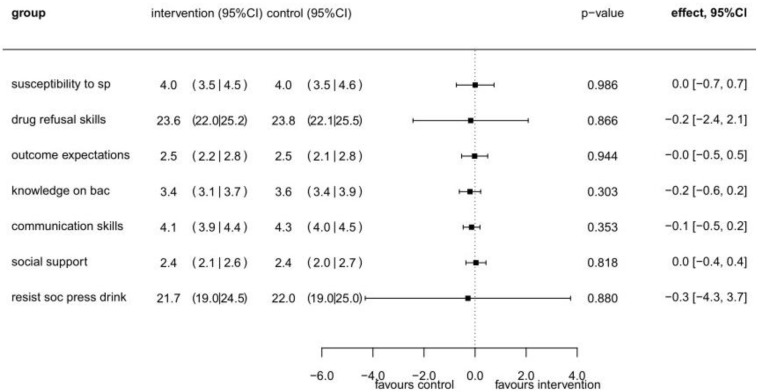
Intervention effects for secondary outcomes (drinking refusal self-efficacy, susceptibility to social pressure, drug refusal skills, outcome expectation, knowledge on blood alcohol concentration, communication skills, and social support) * at T2 (*n* = 378), based on linear mixed models ^#^. * Labels used in graph: social pressure: “sp”; blood alcohol concentration: “bac”; drinking refusal self-efficacy: “resist soc press drink”. ^#^ Separate regression models were used for each outcome. The models are adjusted for sex, age, and the particular baseline value.

At the second follow-up (T2) we found no substantial differences between the intervention and control group in terms of secondary outcomes (see Figure 4).

### 3.7. Adverse Effects

Table 4 depicts information about the potential adverse effects of the intervention and control session. The majority of students in the intervention and control did not experience any adverse effects from participating in the study (293/372, 78.8%). Among the students who experienced adverse effects, symptoms of cybersickness were the most prevalent adverse effect (65/372, 17.5%). 

No substantial differences in the number of adverse effects between the intervention and control groups were observed.

## 4. Discussion

By developing and testing a virtual reality simulation to train alcohol refusal self-efficacy, we entered new grounds in prevention research [21,29]. Our study evaluated the efficacy of the *VR FestLab* game aimed at improving refusal self-efficacy of adolescents who face social pressures to drink. We found a small non-significant effect of drinking refusal self-efficacy as the primary outcome at the first follow-up, but almost no intervention effect at the second follow-up. For all secondary outcomes no substantial differences between the intervention and control groups were found. This certainly requires further exploration, as it is unknown whether this lack of intervention effect is rooted in the program theory [34] regarding the education, training, modelling, and coercion/incentivization elements of the simulation or in its technical realization, and/or the content/characters of the virtual simulation. However, qualitative findings from the pilot testing of *VR FestLab* do not suggest a failure in the intervention design because the users provided positive feedback with regard to many features of the simulation game and would like to explore it further [56]. However, since users steer the gameplay experience, it remains unclear which educational elements the students in the intervention group actually received, and the composition of intervention elements was not uniform for all study participants in the intervention group. Therefore, further analysis is needed to validate the program theory.

Another explanation why we observed no or only minor effects can be the lack of power in the trial design. The sample size at T2 did not fully reach the calculated sample size due to the difficulty of reaching out to schools during the pandemic. In addition, due to the cluster-sampling design small effects are more difficult to detect than in regular RCTs. Therefore, we regard the testing of this prototype as a first and important step towards further development of virtual simulation game-based alcohol and other drug prevention tools, which has been called for by researchers [21].

Our results highlight the small effects that such a brief VR alcohol intervention of limited gameplay followed by in-class discussions can achieve. In this RCT, the exposure was limited as it was only possible to test the app for one period of 15 min. However, *VR FestLab* is designed to be downloaded on the students´ own smartphones to facilitate further exploration in class and/or after school. Therefore, the small effect of a single dose might increase with more frequent usage of the app, which should be studied in future trials.

Although we did not observe a significant effect on the primary outcome, we found interesting tendencies for certain sub-groups. There was a stronger effect for students under the age of 16. Although this observation is in contrast to the conclusion of a review recommending refusal skills programs for older adolescents [19], our findings indicate that *VR FestLab* should be used in class grades below the grades we approached in this RCT. Furthermore, *VR FestLab* tends to have a stronger effect on the drinking refusal self-efficacy of girls. This can be explained by the gender differences seen in gameplay where males prefer more action-oriented games than females [57], and action or competition has not been a priority in the design of *VR FestLab* [32]. Further, in *VR FestLab*, the interaction with peers is a core component and research found that women are more active in seeking friendships in online games [57], which may explain our finding of stronger effects among girls. Finally, we identified that there was a stronger effect for students with low/medium family affluence compared with those with high family affluence. We cannot explain this finding, but we regard it as promising for the use of VR in drug education, because adolescents from low affluence families are generally harder to reach in prevention programs [58].

An important finding of our study is that despite our results being small, the observed effects went in the direction of the study’s hypothesis. Therefore, our results indicate that the intervention does not produce counterproductive effects. This is an important finding, because potential negative impacts of the usage of technology need to be taken into account [59] and simulating a house party may potentially induce positive outcome expectations with respect to alcohol use. Additionally, this finding corresponds to previous mixed methods research of the user experiences of *VR FestLab*, which identified that students experience *VR FestLab* very positively and praised it as a safe environment to explore careful and risk-taking game choices regarding alcohol intake [56]. Further, this study identified that students found the simulation to be realistic and some even reported emotional reactions during gameplay [56]. This is important since previous research [60] found that technological tools often cannot capture the relational and emotional aspects although these are important elements of education.

Utilizing new technologies such as virtual reality has longstanding issues with unwanted adverse effects such as cybersickness [36,61] and there has been increased concern about its safety. The design of this product must take this into consideration [37]. Our results revealed that there was no indication of more adverse effects in the intervention group and the observed adverse effects in both intervention and control group were mild, which is a very positive and reassuring result. To overcome adverse effects, we explicitly stated in writing and verbally to the students that they should discontinue use if any problems should occur.

There are some limitations in this randomized controlled trial. Blinding was not possible due to the nature of the intervention. Although substantial attrition occurred only from T1 to T2, we cannot rule out that attrition affected the power of the analysis. We do not consider a potentially induced bias as very relevant because the attrition was mainly due to non-matching codes and less due to loss-to-follow-up. In addition, the follow-up period was limited to 6 weeks after playing the game and the long-term effects could not be studied. We did not opt for a longer follow-up because *VR FestLab* was developed for repeated use as a free smartphone app, which enables fresh up or even boost up effects. Additionally, the trial is a first testing of this prototype and testing longer term effects is not advised at this early stage. In the same line of argument, we regard the testing of the prototype in the schools of a single region in Denmark as a sufficient starting point, but we cannot fully rule out that adolescents in other regions of the country may use and experience *VR FestLab* differently. In addition, all outcome measures were self-reported and one of the scales (drug refusal skills) showed a too low internal validity in our sample.

Our study was performed during the COVID-19 pandemic. Data collection took place when schools were open, but possibilities for youth to gather outside school for parties was limited due to an assembly ban [62]. Therefore, students did not have the optimal opportunities to train their newly acquired skills from *VR FestLab*, because private and public parties were limited in this period. This can affect the results of our study.

## 5. Conclusions

We developed one of the first virtual reality alcohol prevention simulation games, *VR FestLab*. Our study demonstrated a small non-significant positive effect on drinking refusal self-efficacy at the first follow-up, which was not present at the second follow-up. For all other outcomes, we found no differences between the intervention and control groups. We conclude that the *VR FestLab* gameplay experience should not be applied as a single dose only, but more frequent use should be encouraged. In addition, the intervention can be used as a door opener in combination with other more evidence-based alcohol prevention interventions, such as social norms interventions. Combining approaches is supported by a systematic review concluding that programs combining social influence with social competence components show better results than single component programs and are effective at preventing drug use [13].

The simulation game *VR FestLab* can be a new contribution to schools’ existing prevention practice regarding alcohol prevention, but more research is needed to test the program theory. Since the simulation game can be downloaded to smartphones without cost, user costs are minimal and limited to Google Cardboard or similar products and to earphones to limit disturbing noise. However, practitioners may still need guidance in planning the educational session supported by *VR FestLab* and training on how to reflect game play experiences with adolescents to best support the preventive learning paths of adolescents.

## Figures and Tables

**Table 2:** Baseline characteristics of the study population stratified by intervention and control group.

	Intervention		Control		Total Cohort	
	(*n* = 183)		(*n* = 195)		(*n* = 378)	
	*n*	%	*n*	%	*n*	%
Sex						
Male	92	50.3	94	48.2	186	49.2
Female	91	49.7	101	51.8	192	50.8
Age						
14	8	4.4	4	2.1	12	3.2
15	63	34.4	90	46.2	153	40.5
16	96	52.5	68	34.9	164	43.4
17	15	8.2	31	15.9	46	12.2
18	1	0.5	2	1.0	3	0.8
Perceived family affluence						
Low to medium ^a^	160	87.4	159	81.5	319	84.4
High ^b^	23	12.6	36	18.5	59	15.6
Lifetime binge drinking						
No	47	25.7	54	27.7	101	26.7
Yes	136	74.3	141	72.3	277	73.3

^a^ Response options “Not at all well-off”, “Not so well-off” and “average” combined. ^b^ Response options “Quite well-off” and “Very well-off” combined.

**Table 3:** Primary and secondary outcomes at T0, T1, and T2 stratified by intervention and control group, mean and standard deviation (SD).

	Intervention						Control					
	T0		T1		T2		T0		T1		T2	
	(*n* = 183)		(*n* = 181)		(*n* = 102)		(*n* = 195)		(*n* = 191)		(*n* = 112)	
	Mean	SD	Mean	SD	Mean	SD	Mean	SD	Mean	SD	Mean	SD
Drinking refusal self-efficacy (Range: 5–30)	20.2	6.7	21.2	6.9	22.3	6.8	20.0	7.0	20.4	7.4	21.5	6.3
Drug refusal skills (Range: 7–28)	22.9	5.4	22.7	5.2	24.0	5.1	22.9	5.3	22.9	5.2	23.3	4.6
Knowledge/awareness of blood alcohol concentration (Range: 2–10)	3.6	0.9	3.6	0.9	3.5	1.0	3.4	1.1	3.5	1.1	3.6	1.0
Communication skills (Range: 2–10)	4.3	0.9	4.2	0.9	4.2	0.9	4.3	0.8	4.2	0.9	4.3	0.7
Social support willingness (Range: 0–4)	2.5	0.9	2.6	0.9	2.5	0.9	2.5	0.8	2.5	0.8	2.4	0.9
Susceptibility to peer pressure (Range: 1–5)	2.7	1.3	2.8	1.3	3.0	1.3	3.2	1.4	3.2	1.4	3.2	1.2
Outcome expectations (Range: 1–5)	2.2	0.9	2.3	0.9	2.4	0.9	2.3	1.0	2.3	0.9	2.4	1.0

**Table 4:** Potential adverse effects experienced by the study population at T1.

	Intervention			Control			Total Cohort		
	(*n* = 181)			(*n* = 191)			(*n* = 372)		
	*n*	%	(95% CI)	*n*	%	(95% CI)	*n*	%	(95% CI)
None	141	77.9	71.2–83.7	152	79.6	73.2–85.1	293	78.8	74.3–82.8
Yes, symptoms of cybersickness	33	18.2	12.9–24.6	32	16.8	11.8–22.8	65	17.5	13.8–21.7
Yes, other symptoms/not specified	4	2.2	0.6–5.6	3	1.6	0.3–4.	7	1.9	0.8–3.8
Yes, physical distress on face from wearing the VR equipment	4	2.2	0.6–5.6	6	3.1	1.2–6.7	10	2.7	1.3–4.9

## Data Availability

The fully anonymized datasets for this study are available from the authors upon reasonable request. For access to the datasets, please contact the principal investigator Christiane Stock (cstock@health.sdu.dk). The type of data that is available upon request is data from the pre, post, and follow-up surveys. All researchers or students in higher education are eligible for access to the data. Accessed data will be available for statistical analysis and will be accessed by being granted access to a cloud where data is stored.

## References

[1] ESPAD Group (2020). ESPAD report 2019: Results from the European School Survey Project on Alcohol and other Drugs.

[2] Meyer M., Lundgaard P., Zachariasen E., Christensen A. (2020). Unges Alkoholvaner i Danmark 2019.

[3] Babor T., Caetano R., Casswell S., Edwards G., Giesbrecht N., Graham K. (2010). Alcohol: No ordinary commodity—A summary of the second edition. Addiction.

[4] Wechsler H. (1994). Health and behavioral consequences of binge drinking in college: A national survey of students at 140 campuses. JAMA.

[5] Hingson R., White A. (2014). New research findings since the 2007. Surgeon general’s call to action to prevent and reduce Underage drinking: A review. J. Stud. Alcohol. Drugs.

[6] Flory K., Lynam D., Milich R., Leukefeld C., Clayton R. (2004). Early adolescent through young adult alcohol and marijuana use trajectories: Early predictors, young adult outcomes, and predictive utility. Dev. Psychopathol..

[7] Grant B.F., Stinson F.S., Harford T.C. (2001). Age at onset of alcohol use and DSM-IV alcohol abuse and dependence: A 12-year follow-up. J. Subst. Abuse.

[8] Jennison K.M. (2004). The short-term effects and unintended long-term consequences of binge drinking in college: A 10-year follow-up study. Am. J. Drug Alcohol. Abuse.

[9] Inchley J., Currie D., Vieno A., Torsheim T., Ferreira-Borges C., Weber M., Barnekow V., Breda J. (2018). Adolescent Alcohol-Related Behaviours: Trends and Inequalities in the WHO European Region, 2002–2014.

[10] Griffin K.W., Botvin G.J. (2010). Evidence-based interventions for preventing substance use disorders in adolescents. Child Adolesc. Psychiatr. Clin. N. Am..

[11] Hendricks G., Savahl S., Florence M. (2015). Adolescent peer pressure, leisure boredom, and substance use in low-income Cape Town communities. Soc. Behav. Personal. Int. J..

[12] Sankar M., Maha M., Padmapriya M. (2020). Peer pressure in alcohol abuse of adolescence. Int. J. Multidiscip Educ. Res..

[13] Faggiano F., Minozzi S., Versino E., Buscemi D., Cochrane Drugs and Alcohol Group (2014). Universal School-Based Prevention for Illicit Drug Use. Cochrane Database Syst. Rev..

[14] Young R.M., Oei T.P.S. (2000). The predictive utility of drinking refusal self-efficacy and alcohol expectancy. Addict. Behav..

[15] Oei T.P.S., Morawska A. (2004). A cognitive model of binge drinking: The influence of alcohol expectancies and drinking refusal self-efficacy. Addict. Behav..

[16] Scheier L.M., Botvin G.J., Diaz T., Griffin K.W. (1999). Social skills, competence, and drug refusal efficacy as predictors of adolescent alcohol use. J. Drug Educ..

[17] Donaldson S.I., Graham J.W., Piccinin A.M., Hansen W.B., Marlatt G.A., VandenBos G.R. (1997). Resistance-skills training and onset of alcohol use: Evidence for beneficial and potentially harmful effects in public schools. Addictive Behaviors: Readings on Etiology, Prevention, and Treatment.

[18] Compton J., Jackson B., Dimmock J.A. (2016). Persuading others to avoid persuasion: Inoculation theory and resistant health attitudes. Front. Psychol..

[19] Onrust S.A., Otten R., Lammers J., Smit F. (2016). School-based programmes to reduce and prevent substance use in different age groups: What works for whom? Systematic review and meta-regression analysis. Clin. Psychol. Rev..

[20] Steinberg L., Monahan K.C. (2007). Age differences in resistance to peer influence. Dev. Psychol..

[21] Durl J., Dietrich T., Pang B., Potter L.E., Carter L. (2018). Utilising virtual reality in alcohol studies: A systematic review. Health Educ. J..

[22] Au E.H., Lee J.J. (2017). Virtual reality in education: A tool for learning in the experience age. Int. J. Innov. Educ..

[23] Barko T., Sadler T.D. (2013). Practicality in virtuality: Finding student meaning in video game education. J. Sci. Educ. Technol..

[24] McGrath D., Wegener M., McIntyre T.J., Savage C., Williamson M. (2010). Student experiences of virtual reality: A case study in learning special relativity. Am. J. Phys..

[25] Dietrich T., Rundle-Thiele S., Kubacki K., Durl J., Gullo M.J., Arli D., Connor J.P. (2019). Virtual reality in social marketing: A process evaluation. Mark. Intell. Plan..

[26] Weser V.U., Duncan L.R., Sands B.E., Schartmann A., Jacobo S., François B., Hieftje K.D. (2021). Evaluation of a virtual reality E-cigarette prevention game for adolescents. Addict. Behav..

[27] Weser V.U., Duncan L.R., Pendergrass T.M., Fernandes C.S., Fiellin L.E., Hieftje K.D. (2021). A quasi-experimental test of a virtual reality game prototype for adolescent E-Cigarette prevention. Addict. Behav..

[28] Norris A.E., Hughes C., Hecht M., Peragallo N., Nickerson D. (2013). Randomized trial of a peer resistance skill-building game for hispanic early adolescent girls. Nurs. Res..

[29] Prediger C., Helmer S.M., Hrynyschyn R., Stock C. (2021). Virtual reality-based alcohol prevention in adolescents: A systematic review. Adolescents.

[30] Hadley W., Houck C.D., Barker D.H., Garcia A.M., Spitalnick J.S., Curtis V., Roye S., Brown L.K. (2014). Eliciting affect via immersive virtual reality: A tool for adolescent risk reduction. J. Pediatr. Psychol..

[31] Rundle-Thiele S., Schuster L., Dietrich T., Russell-Bennett R., Drennan J., Leo C., Connor J.P. (2015). Maintaining or changing a drinking behavior? GOKA’s short-term outcomes. J. Bus. Res..

[32] Lyk P.B., Majgaard G., Vallentin-Holbech L., Guldager J.D., Dietrich T., Rundle-Thiele S., Stock C. (2020). Co-designing and learning in virtual reality: Development of tool for alcohol resistance training. Electron. J. E-Learn.

[33] Vallentin-Holbech L., Guldager J.D., Dietrich T., Rundle-Thiele S., Majgaard G., Lyk P., Stock C. (2020). Co-creating a virtual alcohol prevention simulation with young people. Int. J. Environ. Res. Public Health.

[34] Michie S., Van Stralen M., West R. (2011). The behaviour change wheel: A new method for characterising and designing behaviour change interventions. Implement. Sci..

[35] Stock C., Guldager J.D., Grittner U. (2021). Statistical Analysis Plan: “Efficacy Testing of the VR-Game ‘VR FestLab’—Does a VR Based Game Improve Alcohol Resistance Skills Among Adolescents?”, Figshare. https://figshare.com/articles/online_resource/Statistical_Analysis_Plan/14141114.

[36] Davis S., Nesbitt K., Nalivaiko E. A Systematic Review of Cybersickness. Proceedings of the 2014 Conference on Interactive Entertainment.

[37] Rebenitsch L., Owen C. (2016). Review on cybersickness in applications and visual displays. Virtual Real..

[38] McCauley M.E., Sharkey T.J. (1992). Cybersickness: Perception of self-motion in virtual environments. Presence. Teleoperators Virtual Environ..

[39] Young R.M., Hasking P.A., Oei T.P.S., Loveday W. (2007). Validation of the drinking refusal self-efficacy questionnaire—Revised in an adolescent sample (DRSEQ-RA). Addict. Behav..

[40] Macaulay A.P., Griffin K.W., Botvin G.J. (2002). Initial internal reliability and descriptive statistics for a brief assessment tool for the life skills training drug-abuse prevention program. Psychol. Rep..

[41] Shope J.T., Copeland L.A., Maharg R., Dielman T.E., Butchart A.T. (1993). Assessment of adolescent refusal skills in an alcohol misuse prevention study. Health Educ. Q..

[42] Dielman T.E., Campanelli P.C., Shope J.T., Butchart A.T. (1987). Susceptibility to peer pressure, self-esteem, and health locus of control as correlates of adolescent substance abuse. Health Educ. Q..

[43] Bendtsen P., Mikkelsen S., Tolstrup J. (2015). Ungdomsprofilen 2014: Sundhedsadfærd, Helbred og Trivsel Blandt Elever på Ungdomsuddannelser.

[44] Currie C., Inchley J., Molcho M., Lenzi M., Veselska Z., Wild F. (2014). Health Behaviour in School-Aged Children (HBSC) Study Protocol: Background, Methodology and Mandatory Items for the 2013/14 Survey.

[45] Kraus L., Nociar A. (2016). ESPAD Report 2015—Results from the European School Survey Project on Alcohol and other Drugs.

[46] Hoyle R.H., Stephenson M.T., Palmgreen P., Lorch E.P., Donohew R.L. (2002). Reliability and validity of a brief measure of sensation seeking. Personal. Individ. Differ..

[47] LaViola J.J. (2000). A discussion of cybersickness in virtual environments. ACM SIGCHI Bull..

[48] World Health Organization (2020). Coronavirus Disease 2019 (COVID-19): Situation Report, 5.

[49] Ehret P.J., Ghaidarov T.M., LaBrie J.W. (2013). Can you say no? Examining the relationship between drinking refusal self-efficacy and protective behavioral strategy use on alcohol outcomes. Addict. Behav..

[50] R Core Team (2020). R: A Language and Environment for Statistical Computing. R Foundation for Statistical Computing.

[51] Wickham H., Averick M., Bryan J., Chang W., McGowan L.D.A., François R., Grolemund G., Hayes A., Henry L., Hester J. (2019). Welcome to the Tidyverse. J. Open Source Softw..

[52] van Buuren S., Groothuis-Oudshoorn K. (2011). Mice: Multivariate Imputation by Chained Equations in R. J. Stat. Softw..

[53] Bates D., Mächler M., Bolker B., Walker S. (2014). Fitting Linear Mixed-Effects Models Using lme4. arXiv Prepr..

[54] Lenth R.V. (2021). Emmeans: Estimated Marginal Means, aka Least-Squares Means. R Package Version 1.7.0.

[55] Viechtbauer W. (2010). Conducting Meta-Analyses in *R* with the metafor Package. J. Stat. Softw..

[56] Guldager J.D., Kjær S.L., Lyk P., Dietrich T., Rundle-Thiele S., Majgaard G., Stock C. (2020). User experiences with a virtual alcohol prevention simulation for danish adolescents. Int. J. Environ. Res. Public Health.

[57] Veltri N., Krasnova H., Baumann A., Kalayamthanam N. Gender differences in online gaming: A literature review. Proceedings of the Twentieth Americas Conference on Information Systems.

[58] Wood S., Bellis M. (2017). Socio-Economic Inequalities in Alcohol Consumption and Harm: Evidence for Effective Interventions and Policy Across EU Countries.

[59] Limone P., Toto G.A. (2021). Psychological and emotional effects of digital technology on children in COVID-19 pandemic. Brain. Sci..

[60] Saredakis D., Szpak A., Birckhead B., Keage H.A.D., Rizzo A., Loetscher T. (2020). Factors associated with virtual reality sickness in head-mounted displays: A systematic review and meta-analysis. Front. Hum. Neurosci..

[61] Toto G.A. (2018). From educational contexts to addictions: The role of technology in teaching methodologies and in prevention as an educational function. J. E-Learn Knowl. Soc..

[62] Danish Health Authority (2020). COVID-19 i Danmark, Status Ved Indgang Til 5. Epidemiuge.

